# The Role of Preoperative Chemotherapy in the Management of Synchronous Resectable Colorectal Liver Metastases: A Meta-Analysis

**DOI:** 10.3390/curroncol30050340

**Published:** 2023-04-25

**Authors:** Kostas Tepelenis, Georgios Pappas-Gogos, Panagiotis Ntellas, Konstantinos Tsimogiannis, Katerina Dadouli, Davide Mauri, Georgios K. Glantzounis

**Affiliations:** 1 Hepatobiliary and Pancreatic Surgery (HPB) Unit, Department of Surgery, University Hospital of Ioannina and Faculty of Medicine, School of Health Sciences, University of Ioannina, 451 10 Ioannina, Greece; kostastepelenis@gmail.com (K.T.); gepapas@med.duth.gr (G.P.-G.); ktsimogiannis@gmail.com (K.T.); 2 Second Department of Surgery, General University Hospital of Alexandroupolis and Medical School, Democritus University of Thrace, 691 00 Alexandroupoli, Greece; 3 Department of Oncology, University Hospital of Ioannina and Faculty of Medicine, School of Health Sciences, University of Ioannina, 451 10 Ioannina, Greece; ntellasp@gmail.com (P.N.); dmauri@uoi.gr (D.M.); 4 Laboratory of Hygiene and Epidemiology, Faculty of Medicine, University of Thessaly, 382 21 Larisa, Greece; adadouli@uth.gr

**Keywords:** preoperative chemotherapy, neoadjuvant chemotherapy, synchronous colorectal liver metastases, resectable colorectal liver metastases, stage IV colorectal cancer

## Abstract

Background: The indications of preoperative chemotherapy, for initially resectable synchronous colorectal liver metastases, remain controversial. This meta-analysis aimed to assess the efficacy and safety of preoperative chemotherapy in such patients. Methods: Six retrospective studies were included in the meta-analysis with 1036 patients. Some 554 patients were allocated to the preoperative group, and 482 others were allocated to the surgery group. Results: Major hepatectomy was more common in the preoperative group than in the surgery group (43.1% vs. 28.8%, *p* < 0.001). Furthermore, the percentage of patients with more than three liver metastases was higher in the preoperative group compared to the surgery group (12.6% vs. 5.4%, *p* < 0.002). Preoperative chemotherapy showed no statistically significant impact on overall survival. Combined disease free/relapse survival analysis of patients with high disease burden (liver metastases > 3, maximum diameter > 5 cm, clinical risk score ≥ 3) demonstrated that there is a 12% lower risk of recurrence in favor of preoperative chemotherapy. Combined analysis showed a statistically significant (77% higher probability) of postoperative morbidity in patients who received preoperative chemotherapy (*p* = 0.002). Conclusions: Preoperative chemotherapy should be suggested in patients with high disease burden. The number of cycles of preoperative chemotherapy should be low (3–4) to avoid increased postoperative morbidity. However more prospective studies are needed to clarify the exact role of preoperative chemotherapy in patients with synchronous resectable colorectal liver metastases.

## 1. Introduction

Colorectal cancer (CRC) is a significant health burden representing 10% of all cancers. It is the third most commonly diagnosed cancer in males and the second in females, according to the World Health Organization GLOBOCAN database. CRC is the second cause of cancer-related mortality and accounts for 9.4% of worldwide cancer-related deaths [1].

It is estimated that 50–60% of patients diagnosed with CRC develop liver metastases during their disease course. Unfortunately, 80–90% of these patients are not suitable for potentially curative resection. Synchronous colorectal liver metastases (SCRLM) develop in 20–34% of patients with CRC [2,3,4,5]. Autopsy reports have demonstrated that more than half of patients who died of CRC had liver metastases, while in one-third of patients who died of CRC, the liver was the only site of metastatic disease [5].

Synchronous metastatic liver disease implies worse cancer biology and as expected worse survival than metachronous disease [2,4,6,7]. There is no uniform definition of SCRLM in the literature as it varies regarding the time of liver disease diagnosis compared to the diagnosis of the primary tumor. Definitions include metastases detected at or before the primary tumor, while others encompass metastases detected up to three or even six months after the diagnosis of the primary tumor [3,6]. The EGOSLIM consensus group classified colorectal liver metastases (CRLM) as synchronous (detected at or before the diagnosis of the primary tumor), early metachronous (detected within 12 months after diagnosis or surgery of the primary tumor), and late metachronous (detected more than 12 months after diagnosis or surgery of the primary tumor) [6].

Liver resection is the only potentially curative treatment of CRLM. Patients with CRLM who underwent hepatectomy displayed a prolonged survival with a reported 5-year and 10-year survival rates of 30–60% and 25%, respectively [3,4,7,8,9]. A multi-center trial from the Netherlands has shown a large inter-hospital and inter-regional variation in the utilization of liver resection for patients with synchronous colorectal liver metastases. Patients diagnosed with SCLM in expert centers had a higher chance of undergoing liver resection [10].

The main disadvantage of liver resection is the high rate of relapse, which in several studies is as high as 50% [8]. Several clinical risk scores have been developed to predict prognosis after liver resection. These scoring systems stratify patients with CRLM into different risk groups [11,12,13,14]. The most widely used preoperative oncological score was developed by Fong et al. Five factors were found to be significant and independent predictors of poor long-term outcome: disease-free interval <12 months, number of metastases > 1, preoperative carcinoembryonic antigen >200 ng/mL, largest liver metastasis >5 cm and lymph node-positive primary tumor. Patients with up to two criteria are associated with a favorable outcome (low risk), while patients with three or more criteria are at increased risk of recurrence (high risk) [11].

In recent years, preoperative chemotherapy (PREC) has been widely used for the management of CRLM, both synchronous and metachronous liver deposits. The theoretical benefits of PREC encompass the evaluation of chemo-responsiveness, shrinkage of the metastases and eradication of the micro-metastases [2,3,8]. Potential drawbacks include sinusoidal obstruction syndrome, steatosis and disease progression. Generally, the extent of liver damage depends on the type and duration of the chemotherapy [7].

The role of PREC in initially resectable synchronous colorectal liver metastases remains controversial. The National Comprehensive Cancer Network recommends the following options for patients with initially resectable SCRLM: (1) synchronous or staged colectomy with liver resection followed by adjuvant chemotherapy; (2) preoperative chemotherapy for 2 to 3 months followed by synchronous or staged colectomy with liver resection, then adjuvant chemotherapy; (3) colectomy followed by chemotherapy and a staged resection of metastatic disease, then adjuvant chemotherapy [5]. Several studies failed to demonstrate any overall survival benefit of patients with initially resectable SCRLM receiving PREC [15,16,17,18,19,20,21]. Only one of these studies reported a higher relapse-free survival in patients who received PREC [17]. Moreover, a subgroup of patients receiving PREC experiences disease progression during treatment. Responders displayed an improved overall survival compared to non-responders^16^ or patients not receiving PREC [14].

This meta-analysis aimed to assess the efficacy and safety of PREC prior to liver resection in patients with initially resectable SCRLM. A subgroup analysis of patients at increased risk of recurrence was also performed.

## 2. Materials and Methods

This systematic review and meta-analysis were conducted following the Preferred Reporting Items for Systematic Reviews and Meta-Analyses (PRISMA) statement (PROSPERO id 411865) [22].

### 2.1. Literature Search

PubMed and Embase were searched from 1 January 2000 until 30 June 2021. The keywords: preoperative chemotherapy, neoadjuvant chemotherapy, synchronous colorectal liver metastases, resectable colorectal liver metastases and stage IV colorectal cancer were selected to identify all reports possibly related to the role of preoperative chemotherapy in the management of synchronous resectable colorectal liver metastases. Reference lists of all relevant studies and reviews were scanned further for additional studies. Study authors were contacted for unpublished studies or additional data.

### 2.2. Eligibility Criteria

Studies examining patients with synchronous resectable colorectal liver metastases aged 18 years or older were eligible. Different definitions of synchronous metastases can be found in the literature, and the adoption of a standardized definition is needed to clarify future reporting. In the present study, liver metastases were considered synchronous if detected on radiographic imaging in the perioperative period, intraoperatively during primary tumor resection or in the first 3 postoperative months. High disease burden was defined as patients with a Clinical Risk Score ≥3 [19], with a maximum diameter > 5 cm [16] or patients with more than three metastases [20].

All study designs, i.e., randomized control trials, cohort studies, case-control studies and case series examining the efficacy of preoperative chemotherapy in the management of synchronous resectable colorectal liver metastases, were included. Abstracts or conference communications not published as full articles in peer-reviewed journals were excluded. Additionally, review articles, case reports and editorials were ruled out. Restrictions were imposed on the publication date and language.

The patients were classified into two groups: those who received preoperative chemotherapy prior to liver resection and those who underwent upfront liver resection.

### 2.3. Outcomes

The primary outcomes were survival (OS) and disease-free survival (DFS). A subgroup analysis of patients with a high disease burden was also performed. Secondary outcomes encompassed R0 resection and morbidity.

### 2.4. Study Selection

After pilot testing eligibility criteria for citations and full-text articles, two reviewers screened the search results independently to select potentially eligible records based on title and abstract. Subsequently, the two reviewers confirmed the eligibility criteria independently based on the full-text articles of the relevant selected papers. Disputes were settled by discussion. If no agreement could be reached, a third reviewer would decide.

### 2.5. Data Collection and Extraction

The exact process was followed for data extraction. If needed, authors were contacted for studies including unclear or missing data. The two reviewers extracted information regarding characteristics of the study population (sex, mean age), primary tumor location (colon or rectal cancer), primary tumor size (T stage), lymph node status (N stage), liver metastases’ features (solitary or not, unilateral or bilateral, number of liver metastases >3, the diameter of the largest metastasis >5 cm), presence of extrahepatic disease, type of chemotherapy, response to chemotherapy, type of surgery, R0 resection, overall survival, disease-free survival, mortality, morbidity, recurrence rate and modality of recurrence. Patients were classified into two groups based on intervention: preoperative chemotherapy followed by surgery (preoperative group) and upfront surgery followed by chemotherapy (surgery group). The preoperative chemotherapy was divided into oxaliplatin-based, irinotecan-based and chemotherapy plus biological agents. The surgical approach was also divided into three categories: simultaneous primary tumor and liver metastases resection (simultaneous), primary tumor first approach (colon first) and liver first approach (liver first). Patients with a high disease burden were defined as patients with a Clinical Risk Score ≥ 3 [19], patients with more than three metastases or with a maximum diameter > 5 [16,20]. Disputes were resolved by discussion. The inclusion criteria were the following:

P(opulation) = adults with synchronous resectable colorectal liver metastases.

I(ntervention) = preoperative chemotherapy followed by surgery, and surgery followed by chemotherapy as control.

C(omparison) = all types of comparisons were included.

O(utcomes) = overall survival, disease-free survival (primary outcomes), R0 resection, and morbidity (secondary outcomes). A subgroup analysis of patients with high disease burden regarding overall and disease-free survival was also performed.

S(tudy design) = Any study design, i.e., randomized control trials, cohort studies, case-control studies.

### 2.6. Statistical Analysis

Categorical variables are described with the use of frequency and continuous variables with medians. Categorical data were analyzed with the use of Chi-square test and continuously using the Mann–Whitney U test.

Summary data methodology meta-analysis of available trials was used for the analyses. The Inverse-Variance (IV) and the Mantel–Haenszel (MH) statistical methods were applied to calculate pooled HRs and ORs. Among studies, heterogeneity was evaluated with Cochran’s Q test; in the case of statistically significant heterogeneity (Q test *p* < 0.1), the Random Effects (RE) model was reported; otherwise, the Fixed Effects (FE) model was adopted to estimate the pooled ratios. I2-statistic was also calculated to assess overall heterogeneity. Some 95% confidence intervals (CIs) were used for the analysis. Statistical significance was set at the two-sided 0.05 level. Rev Man software V5.4 was used to complete the pooled data analysis.

Meta-regression was performed in IBM SPSS V29 to control for confounding variables and identify the independent predictors of disease-free survival and overall survival. Funnel plot and Egger’s test [23] were used to assess publication bias. Egger’s test was conducted in IBM SPSS V29. In the studies where HR and 95% CI were not directly provided from the published data, we estimated them according to the methods indicated by Parmar et al. [24] and Tierney et al. [25]. Where necessary, the published Kaplan–Meier survival curves were also utilized to reconstruct the data needed for calculating the HR and OR along with their associated 95% CI. For this reason, digitizing software (Get Data Graph Digitizer) was employed to extract the data from the Kaplan–Meier survival curves with the highest precision possible.

## 3. Results

### 3.1. Study and Patients’ Characteristics

**Figure 1:**
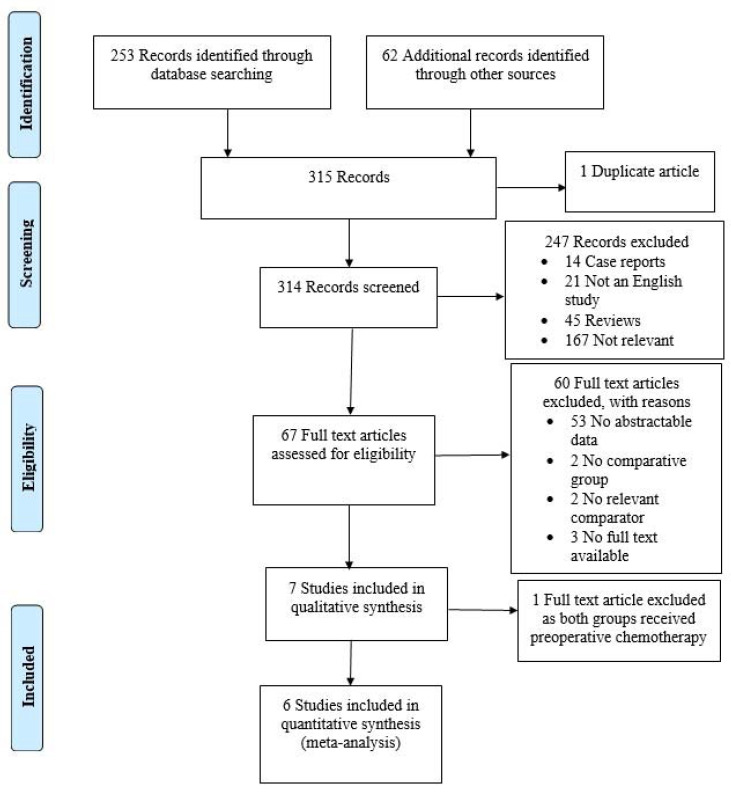
Flow diagram of study selection.

A total of 67 articles were identified through a database search. The full text of 64 articles was retrieved and examined in more detail, while the full text of the remaining three articles was not obtainable. The authors of those three articles were contacted, but unfortunately, no one responded to our query. As a result, these articles were ruled out. Fifty-seven articles were also excluded as they contained no extractable data (53 articles), no comparative group (two articles) or no relevant comparator (two articles). Seven studies were included in the qualitative synthesis. One study was excluded as both groups received preop chemo; six studies matched the selection criteria and were suitable for meta-analysis. Figure 1 illustrates the selection process.

The characteristics of the included studies are shown in Table 1. Six retrospective studies were included in the meta-analysis [15,16,17,18,19,20]. Three were multicenter (50%) [16,19,20], and the other three were single-center studies (50%) [15,17,18]. Most articles were conducted in East Asia (50%) [16,17,18], followed by the USA (33.3%) [15,20] and Europe (16.7%) [19]. Half of the studies were published before 2015 (50%) [15,18,20], and the remaining half were published after 2015 (50%) [16,17,19]. The number of patients included per study ranged from 30 to 499. Four studies included 100–199 patients (66.6%) [15,16,17,19], while one study included < 100 patients (16.7%) [18], and one study >400 patients (16.7%) [20].

The patients’ characteristics of the included studies are summarized in Table 2. Of the 1036 patients with synchronous resectable colorectal liver metastases 59.5% were male, and the median age was 59.1 years. A total of 554 were allocated to the preoperative group, and 482 were allocated to the surgery group. Patients in the preoperative group (57% male, median age 57.9 years) received preoperative chemotherapy. They then underwent liver resection, while patients in the surgery group (62.2% male, median age 60.6 years) underwent upfront liver resection and received postoperative chemotherapy. The primary tumor was usually colon cancer (74.5% vs. 63.9%) and was more often categorized as T3/4 (76.4% vs. 77.8%) in both groups. Metastatic disease was found within the regional lymph nodes in 69.7% of preoperative and 63.1% of patients in the surgery group. Simultaneous resection of the primary lesion and liver metastases was performed in 26.7% of patients in the preoperative group and in 54.6% in the surgery group. Major hepatectomy was more common in the preoperative group than in the surgery group (43.1% vs. 28.8%) *p* < 0.001. Furthermore, the percentage of patients with more than three liver metastases was higher in the preoperative group compared to the surgery group (12.6% vs. 5.4%) *p* = 0.002.

### 3.2. Overall Survival

**Figure 2:**
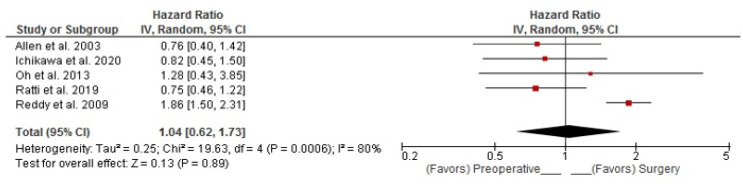
Polled Hazard Ratio (HR) for overall survival (OS). Allen et al. [15], Ichikawa et al. [16], Oh et al. [18], Ratti et al. [19], Reddy et al. [20].

**Figure 3:**
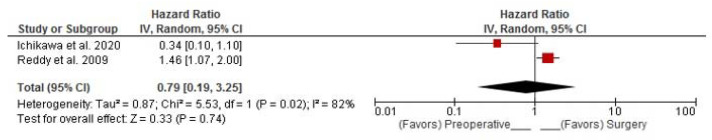
Overall survival (OS) for patients with high disease burden. Ichikawa et al. [16], Reddy et al. [20].

Five RSs evaluated overall survival (OS) [15,16,18,19,20]. Combined analysis showed that PREC did not have a statistically significant impact on OS. (Random Effects pooled HR: 1.04; 95%CI: 0.62–1.73; *p* = 0.890) (Figure 2). Combined OS analysis of patients with high disease burden demonstrated no statistically significant difference between the two treatment strategies in these patients (Random Effects pooled HR: 0.79; 95%CI: 0.19–3.25; *p* = 0.740) (Figure 3).

### 3.3. Disease-Free Survival

Two RSs reported the incidence of disease-free survival (DFS) in the preoperative and surgery group [17,19], while three RSs reported relapse-free survival (RFS) in the preoperative and surgery group [16,18,20]. Since tumor recurrences may be estimated with DFS or RFS by the investigator’s choice, DFS data were analyzed along with RFS data in our study.

**Figure 4:**
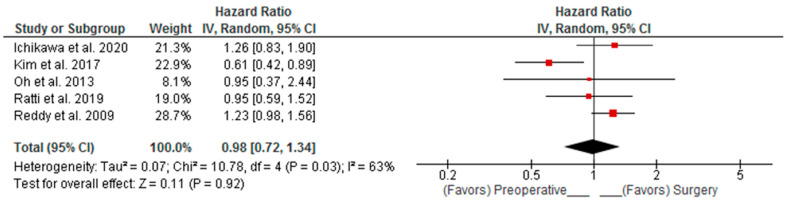
Pooled Hazard Ratio (HR) for disease free survival (DFS) and relapse free survival (RFS). Ichikawa et al. [16], Kim et al. [17], Oh et al. [18], Ratti et al. [19], Reddy et al. [20].

**Figure 5:**
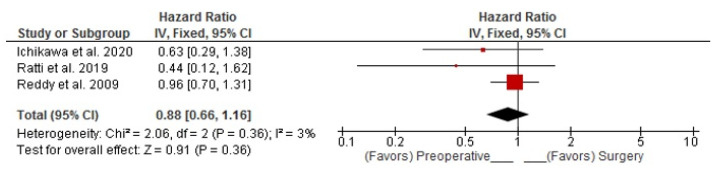
Risk of recurrence for patients with high disease burden. Ichikawa et al. [16], Ratti et al. [19], Reddy et al. [20].

Combined DFS and RFS analysis showed that PREC did not significantly affect recurrence (Random Effects pooled HR: 0.98; 95%CI: 0.72–1.34; *p* = 0.920) (Figure 4). Combined DFS/RFS analysis of patients with high disease burden demonstrated that in these patients, there is a 12% lower risk of recurrence in favor of preoperative chemotherapy; however, this was not statistically significant. (Fixed Effects pooled HR: 0.88; 95%CI: 0.66–1.16; *p* = 0.360) (Figure 5).

### 3.4. R0 Resection

**Figure 6:**
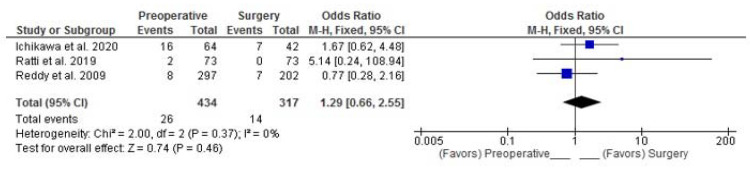
Pooled OR for positive hepatic resection margin. Ichikawa et al. [16], Ratti et al. [19], Reddy et al. [20].

R0 resection was assessed by 3 RSs [16,19,20]. Combined analysis showed that patients in the preoperative group had a 29% higher probability of positive hepatectomy margins; however, this was not statistically significant (Fixed Effects, pooled-OR: 1.29; 95%CI: 0.66–2.55; *p* = 0.46), meaning that the rate of R0 resections between the two groups did not differ significantly (Figure 6).

### 3.5. Morbidity

**Figure 7:**
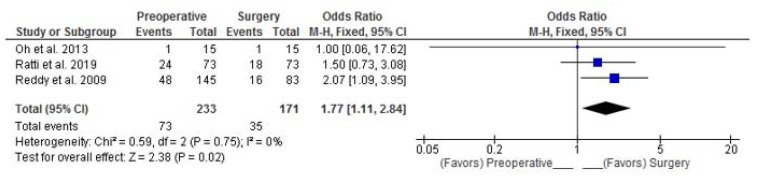
Pooled OR for postoperative morbidity. Oh et al. [18], Ratti et al. [19], Reddy et al. [20].

Three RSs reported the morbidity rate in the preoperative and surgery groups [18,20]. Combined analysis showed a statistically significant 77% higher probability of postoperative morbidity in patients who received PREC (Fixed Effects, pooled-OR: 1.77; 95%CI: 1.11–2.84; *p* = 0.002) (Figure 7).

### 3.6. Recurrence Rate

Three RSs [16,18,19] and 1 RCT [21] reported a recurrence rate of 45.2–64.3% in the preoperative group and 47.9–68.8% in the surgery group. Two studies evaluated the modality of recurrence. Intrahepatic recurrence ranged between 42.4 and 66.7% in the preoperative group and between 45.7 and 77.8% in the surgery group, while extrahepatic recurrence varied between 11.1 and 21.2% in the preoperative and between 17.1 and 22.2% in the surgery group. Finally, concurrent intrahepatic and extrahepatic recurrence occurred in 22.2–36.4% in the preoperative group and 0–37.2% in the surgery group [18,19].

### 3.7. Meta-Regression

Finally, meta-regression analyses were performed with a random-effects model using restricted maximum likelihood estimation (RMLE). Results of meta-regression analysis suggested that none of independent variables (age, N Stage and T Stage) were statistically significant predictors of the relationship between clinicopathological factors and Overall Survival (age: HR = 0.79, *p* = 0.789; N Stage: HR = 2.09, *p* = 0.950; T Stage: HR = 1.46 *p* = 0.951).

## 4. Discussion

Our meta-analysis could not show any statistically significant difference of PREC vs surgery as far as the outcomes are concerned. We could see a trend in DFS with 12% lower risk of recurrence in favor of preoperative chemotherapy in the group with high-burden disease; however, this was not statistically significant. Interestingly enough, we could see a 29% higher probability of positive hepatectomy margins in the preoperative chemotherapy group; however, this was not statistically significant either. Interestingly, the higher R1 numbers did not affect the overall outcomes of this group. A possible explanation is that adjuvant chemotherapy compensates for the R1 margin and that R1 resection itself is not due to bad surgical technique but rather a potent indicator of aggressive tumor biology [26].

As we can see in Table 2, patients in the PREC group had higher tumor burden as reflected by the higher percentage of patients with more than three liver metastases, the higher percentage of major hepatectomies and the lower percentage of simultaneous resections of the primary tumor and liver metastases. As we have similar outcomes in both groups, we could suggest that preoperative chemotherapy can potentially be of benefit.

A notable benefit, but still not statistically significant, could be seen in patients with more than three liver metastases, having a 12% lower risk of recurrence. Despite the higher postoperative morbidity in PREC group, the lower recurrence risk cannot be overlooked, so we can suggest PREC in these patients.

This finding can be supported by the European Organization for Research and Treatment of Cancer (EORTC) study 40983 which has shown that neoadjuvant chemotherapy (in initially resectable metastases) could reduce the risk of relapse by one-quarter and allows the testing of the chemosensitivity of cancer to help to determine the appropriateness of further treatments and to observe progressive disease, which contraindicates immediate surgery [27].

Furthermore, one recent retrospective clinical study with 102 patients from Japan has shown that in patients with resectable colorectal liver metastases, the patients with high tumor burden score (TBS) had a survival benefit from preoperative chemotherapy, while the patients with low TBS score benefited from adjuvant chemotherapy. In multivariate analysis, preoperative chemotherapy was an independent prognostic factor for favorable overall survival only in the TBS-high group [9].

The TBS reported recently by Sasaki et al. is a newly developed model that translates the size and the number of liver metastases into one variable using the Pythagorean theorem, and it seems to have better prognostic discriminatory power than traditional tumor morphologic categorization [28].

Another recent study also coming from Japan agrees with the results of our meta-analysis. A retrospective analysis of a multi-institutional cohort of 222 patients showed that the surgical failure-free survival in the sub-group of 53 patients with synchronous liver metastases and high tumor burden (≥5 lesions and/or maximum tumor diameter > 5 cm) was significantly better in the preoperative chemotherapy group compared to the upfront surgery group [29]. A multicenter retrospective trial showed that patients with high clinical risk score (3–5) benefited from neo-adjuvant chemotherapy [30]. A prospective randomized multicenter clinical trial investigating the role of neo-adjuvant chemotherapy followed by surgery vs. surgery alone in high-risk patients with resectable liver metastases is underway, but the results are not yet available [31].

Unfortunately, our question has not been clearly answered by our meta-analysis. We could only find low quality studies, leading to low evidence. We had a few studies with high heterogeneity in their design which reduced quality.

## 5. Conclusions

In conclusion, based on current evidence, we could suggest PREC in patients with high disease burden, but we need to take into consideration the higher morbidity risk. Considering the low to moderate level of evidence our findings urge the need for high-quality, prospective studies investigating the role of PREC in initially resectable liver metastases. 

## Figures and Tables

**Table 1:** Summary of study characteristics.

Author	Publication Year	Continent *	Study Design	Site	Number of Patients	Definition of Synchronous Colorectal Liver Metastases
Allen et al. [15]	2003	USA	RS **	Single	106	Diagnosed until 1 month postoperatively
Reddy et al. [20]	2009	USA	RS **	Multicenter	499	Diagnosed with the primary tumor in place or during primary tumor resection
Oh et al. [18]	2013	East Asia	RS **	Single	30	Diagnosed with the primary tumor in place
Kim et al. [17]	2017	East Asia	RS **	Single	149	Diagnosed with the primary tumor in place
Ratti et al. [19]	2019	Europe	RS **	Multicenter	146	Diagnosed with the primary tumor in place
Ichikawa et al. [16]	2020	East Asia	RS **	Multicenter	106	Diagnosed with the primary tumor in place

* Continent refers to where the study was conducted; if not reported explicitly, the location of the first author’s institution was used as a proxy. ** RS = Retrospective Study.

**Table 2:** Patients’ characteristics.

Variables	All (*n* = 1036)	Preoperative Group (*n* = 554)	Surgery Group (*n* = 482)	Sig.
Male	59.5%	57%	62.2%	0.089 ^C^
Female	40.5%	43%	37.8%	
Median age (years)	59.1	57.9	60.6	0.485 ^M-W^
Colon cancer	69.6%	74.5%	63.9%	<0.001 ^C^
Rectal cancer	30.4%	25.5%	36.1%	
T stage:				
T1/2	23%	23.6%	22.2%	0.592 ^C^
2. T3/4	77%	76.4%	77.8%	
N stage:				
Negative	33.4%	30.3%	36.9%	0.025 ^C^
2. Positive	66.6%	69.7%	63.1%	
Bilateral liver metastases:				
Yes	8.1%	7.8%	8.5%	0.728 ^C^
2. No	8.9%	8.1%	9.8%	
3. NR	83%	84.1%	81.7%	
Number of liver metastases > 3:				
Yes	9.3%	12.6%	5.4%	0.002 ^C^
2. No	41.8%	43.7%	39.6%	
3. NR	48.9%	43.7%	55%	
Size of the largest metastasis > 5 cm				
Yes	3.7%	4%	3.3%	0.298 ^C^
2. No	7.1%	6.3%	7.9%	
3. NR	89.8%	89.7%	88.8%	
CRS ≥ 3				
Yes	3.4%	3.4%	3.4%	0.641 ^C^
2. No	13.6%	12.5%	14.9%	
3. NR	83%	84.1%	81.7%	
Biological agent:				
Yes	7.1%	13.4%	-	-
2. No	86.4%	74.5%	-	
3. NR	6.5%	12.1%	-	
Type of Surgery:				
Colon first	60.3%	73.3%	45.4%	<0.001 ^C^ (comparison 1 vs. 3)
Liver first	0%	0%	0%	
Simultaneous	39.7%	26.7%	54.6%	
Major Hepatectomy:				
Yes	36.5%	43.1%	28.8%	<0.001 ^C^
2. No	46.2%	44.6%	48.1%	
3. NR	17.3%	12.3%	23.3%	
Adjuvant chemotherapy				
Yes	58%	46.4%	71.4%	<0.001 ^C^
2. No	42%	53.6%	28.6%	
R_0_ resection:				
Yes	67.8%	72.9%	62.5%%	0.287 ^C^
2. No	4.7%	5.4%	3.3%%	
3. NR	27.5%	21.7%	34.2%	

NR: Non-reported; CRS: Clinical Risk Score for patients with colorectal liver metastases; C: Chi-Square test; M-W: Mann–Whitney U test.

## Abbreviations

CRC:	Colorectal Cancer
CRLM:	Colorectal Liver Metastases
DFS:	Disease-Free Survival
OS:	Overall Survival
PREC:	Preoperative Chemotherapy
RCT:	Randomized Control Trial
RS:	Retrospective Study
SCRLM:	Synchronous Colorectal Liver Metastases
